# An analysis of the subtypes of dengue fever infections in Barbados 2003–2007 by reverse transcriptase polymerase chain reaction

**DOI:** 10.1186/1743-422X-5-152

**Published:** 2008-12-17

**Authors:** M Gittens-St Hilaire, Nicole Clarke-Greenidge

**Affiliations:** 1 University of the West Indies, Faculty of Medical Sciences, Queen Elizabeth Hospital, Martindales Road, St. Michael, Barbados; 2 Leptospira Laboratory, Ministry of Health, Enmore #2, Lower Collymore Rock, St. Michael, Barbados

## Abstract

**Background:**

To perform a retrospective analysis of patients with IgM antibodies to dengue fever infection to determine the serotypes present by molecular techniques. A representative sample (~20%/per year) of patients diagnosed with dengue fever infection were selected based on the detection of IgM antibodies in the acute phase serum sample. RNA was extracted from each sample and reverse transcribed. Following this, the amplicons were electrophoresed and serotyped based on band sizes.

**Results:**

This study consisted of 71 males and 101 females ranging in age from 0 – 50+ yrs giving a total of 172 persons with an average of 34.4 patients per year. Onset averaged 6.9 days ranging from 0–90 days. Common symptoms were as follows: fever (69%), headache (52%), arthralgia (36%), ocular pain (32%), emesis (15%) and lumbar pain (15%). All patients investigated with the exception of one, were infected with DENV-3.

**Conclusion:**

DENV-3 is currently circulating on the island and not DENV-1 or DENV-2 as in previous years. This has implications for the enhancement of clinical, laboratory and environmental surveillance systems.

## Background

Dengue is a homonym for the African *ki denga pepo*, which appeared in English literature during an 1827–28 Caribbean outbreak. The first definite clinical report of dengue is attributed to Benjamin Rush in 1789, but the viral aetiology and its mode of transmission via mosquitoes were not established until the early 20th century [1].

Dengue has been called the most important mosquito-transmitted viral disease in terms of morbidity and mortality occurring in most tropical and subtropical regions. Dengue fever is currently endemic in over 100 tropical and non-tropical countries, and imported cases have been reported in several non-endemic countries. The major disease burden occurs in South East Asia, the Americas and the western Pacific. Four serotypes of dengue virus are transmitted corresponding to a geographical area of between 35°N and 35°S latitude where there is the distribution of *A. aegypti*, the principal mosquito vector. *Aedes albopictus, Aedes polynesiensis*, and other species can transmit the virus in specific circumstances [2]. The annual incidence of dengue fever and dengue hemorrhagic fever (DHF) has increased dramatically around the world in recent decades [3,4]; the World Health Organization (WHO) estimates that over 2.5 billion people are currently at risk from dengue viruses globally [5].

Classic dengue fever is an acute febrile disease with headaches, musculoskeletal pain and rash, but the severity of illness and clinical manifestations vary with age. Infection is often asymptomatic or non-specific consisting of fever, malaise, pharyngeal infection, upper respiratory symptoms, and rash – particularly in children. Classic dengue primarily occurs in nonimmune, nonindigenous adults and children. After an incubation period of 4 to 7 days, fever, often with chills, severe frontal headache, and retro-orbital pain-develops abruptly with a rapid progression to prostration, severe musculoskeletal and lumbar back pain, and abdominal tenderness. DHF is a more serious clinical entity. DHF/DSS usually occurs during a second dengue infection in persons with pre-existing actively or passively (maternally) acquired immunity to a heterologous dengue virus serotype. Illness begins abruptly with a minor stage of 2–4 days' duration followed by rapid deterioration. Increased vascular permeability, bleeding, and possible DIC may be mediated by circulating dengue antigen-antibody complexes, activation of complement, and release of vasoactive amines. In the process of immune elimination of infected cells, proteases and lymphokines may be released and activate complement coagulation cascades and vascular permeability factors. In 20–30% of DHF cases, the patient develops shock, known as the dengue shock syndrome (DSS). Worldwide, children younger than 15 years comprise 90% of DHF subjects; however, in the Americas, DHF occurs in both adults and children. There is no real 'safe' season, although there seems to be a cyclical pattern and a rise in infections during rainy seasons [5]. Rising rainfall in some regions has contributed to an extension of the season in recent years.

With the increasing frequency of dengue outbreaks and concurrent circulation in the Caribbean region of all serotypes, places the Caribbean populations at risk for DHF/DSS. Dengue haemorrhagic fever was recorded for the first time in Trinidad in 1992–1993, while in 1995; Jamaica recorded 108 cases of DHF and 3 cases of DSS, with a total of 4 deaths.

Large outbreaks of dengue occurred in Barbados in 1995 and 1997 and were associated with circulation of serotype 1 (1995) and serotype 2 (1997), placing the population at increased risk of DHF. Dengue haemorrhagic fever was first detected in Barbados in 1995 and five fatalities due to DHF occurred in 1997 [6].

This study sought to determine the subtypes of dengue virus circulating in the island over the last five years by molecular techniques and so assess the efficacy of this method in the adaptation of the current investigation protocol to facilitate rapid turnaround times in patient care.

## Results

The total number of requests fluctuated over the last five years ranging from 775 (2003) to 434 (2006) which correlated with the number of cases (presence of IgM antibodies). It should be noted that the number of persons with IgG antibodies outnumbered those with IgM antibodies and the number of new cases was proportional to the number of dengue fever requests. (Table 1)

**Table 1:** Dengue cases per year in Barbados tested for IgM and IgG antibodies, 2003–2007

	2003	2004	2005	2006	2007
Total requests	775	450	501	434	550
IgM +	454	195	78	153	219
IgG +	613	342	376	320	375
IgM-, IgG-	78	72	66	66	94
IgM + only	84	37	3	41	48
Serotype	3	3	3	3	3>2

**Figure 1:**
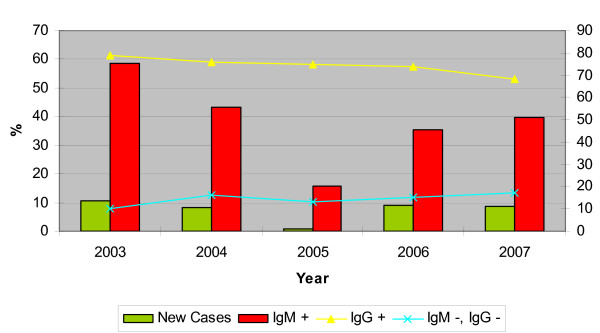
Percentage of dengue cases 2003–2007.

In 2003, a diagnosis of dengue fever was confirmed in 454 (58.6%) of 775 patients by having dengue IgM antibodies. There is an average positivity of 38.5% in patients suspected of having dengue fever over the past five years. However, this figure dipped in 2005, where an average detection rate of 15.6% was obtained over the 12 months of that year (Fig 1).

On average, 34.4 persons were selected per year retrospectively for the study with six seronegative control specimens included (Table 2). It should be noted that these patients were serologically (IgM positive) identified as being dengue positive and selected for confirmation and serotyping by reverse transcriptase polymerase chain reaction. In 2003, 45 specimens were analysed of which 14 were males and 31 were females. There were 22, 22, 22 and 61 patients selected from 2004–2007 respectively (Table 3). This gave a total of 71 males and 101 females included in the study. An average of 20% of the total number of cases selected per year for analysis. Twenty-two patients had unknown dates of onset, however the average duration of onset was 6.88 days with a range of 0 – 90 days. Symptoms included fever (68.5%), headache (52.4%), vomiting (10%), arthralgia (36.2%), retroorbital (ocular) pain (31.5%), jaundice (8.9%), cough (8.9%), chills (12.1%), emesis (15.3%), myalgia (10.5%), diarrhoea (9.7%), lumbar pain (14.5%), rash (12.9%), malaise (12.1%) and thrombocytopenia (3.2%) (Table 4).

**Table 2:** Percentage of patients selected per year for the study based on the presence of IgM antibodies

Year	Cases	No. of cases selected	% of cases selected
2003	454	45	11
2004	195	22	11
2005	79	22	28
2006	153	22	14
2007	219	61	35
Average	220	34.4	20

**Table 3:** Specimens tests prospectively and retrospectively by RT-PCR

Year	Sex	0–16	17–20	21–30	31–40	41–50	50+	Age Unknown	Total	Total (male & female)
**2003**	Male	2	1	2	4	2	1			
	Female	5	3	8	6	2	3	3	31	
**2004**	Male	1	0	2	3	1	2	1	10	**22**
	Female	4	0	2	0	0	1	5	12	
**2005**	Male	1	0	3	3	1	1	1	10	**22**
	Female	0	2	3	2	3	0	2	12	
**2006**	Male	1	1	0	0	1	1	1	5	**22**
	Female	3	2	3	2	1	1	5	17	
**2007**	Male	16	3	6	5	3	3	1	32	**61**
	Female	14	2	7	3	0	4	1	29	
**Total**	Male	21	5	13	15	8	8	6	71	**172**
	Female	26	9	23	13	6	10	16	101	
**Total**		**47**	**14**	**36**	**28**	**14**	**18**	**22**	**172**	

**Table 4:** List of clinical signs and symptoms in patients tested

Clinical/Laboratory feature n = 124	Number of cases	Percentage (%)
Pyrexia	85	69
Arthralgia	45	63
Headache	65	52
Ocular pain	39	32
Emesis	19	15
Lumbar pain	18	15
Rash	16	13
Chills	15	12
Malaise	15	12
Myalgia	13	11
Diarrhoea	12	10
Jaundice	11	9
Cough	11	9
Abdominal pain	8	7
Thrombocytopenia	5	4
Neck pains	5	4
Haematuria	4	3
Hepatitis	4	3
Sore throat	4	3
Bleeding gums	3	2
Blood-shot eyes	1	0.8
Petechiae	1	0.8
Shock	1	0.8

**Figure 2:**
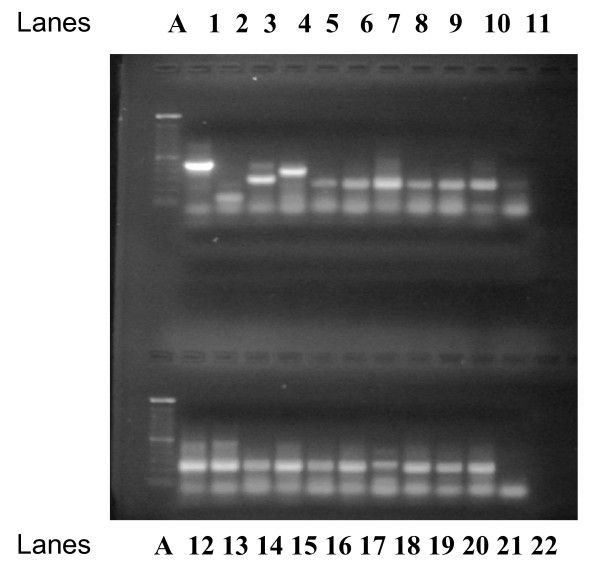
RT-PCR detection and typing of dengue virus in serum from patients infected during the period of 2003–2007 in Barbados. RNA was extracted from serum samples and was amplified by the two-enzyme single tube RT-PCR assay as described in the Materials and Methods section. Lane 1: Dengue 1 RNA (positive control); Lane 2: Dengue 2 RNA (positive control); Lane 3: Dengue 3 RNA (positive control); Lane 4: Dengue 4 RNA (positive control), Lane 5 – 21: patients (DENV- 3 positive); Lane 22: negative control (water): Lane A: 100 bp DNA ladder.

Based on the molecular analysis of all patients, dengue type 3 was recorded for all patients (99.4%) with the exception of 1(0.6%) male who had an infection with dengue type 2 (Fig. 2).

## Discussion

The lack of a vaccine or a cure for dengue fever makes the development of laboratory-based surveillance systems all the more important and to provide essential information for effective vector control programmes. It is of considerable importance to determine the serotypes of circulating dengue virus, when and where since previous infection with one of the four serotypes can be an important risk factor for the development of dengue haemorrhagic fever and dengue shock syndrome (DHF/DSS) upon infection with a heterotypic serotype. The current "gold standard" for typing dengue virus involves isolation of the virus in cultured cells or mosquitoes followed by indirect immunofluorescence. However, this requires cell culture facilities or mosquito colonies, which are difficult to maintain in laboratories in many developing countries. Serological techniques such as immunoglobulin M or G enzyme linked immunosorbent assays (ELISA) with a single serum sample does not provide information on the serotype of the virus. However, single-step reverse transcriptase polymerase chain reaction (RT-PCR) detection and typing of dengue virus offers a sensitive, specific, reproducible and rapid alternative that requires only one acute-phase serum sample [7,8].

The diagnosis of dengue fever by this one-step RT-PCR procedure has reduced the amount of time in getting the results to the patient. In comparison with the IgM ELISA or IgG ELISA methods, antibodies are detectable in the range of 5–10 days, whereas viraemia occurs after a few days of onset (1–8 days) and can be readily measured by RT-PCR which is more sensitive and also provides the serotype of the virus.

A previous study on Barbadian patients with dengue who were initially investigated for leptospirosis, indicated that serotypes during that period (1995–1997) where 1, 2 or 4 [9]. The current study investigating patients during the five year span (2003–2007), revealed that the majority (>99%) of cases were caused by serotype 3. This serotype first presented in Belize in 1963 and has subsequently spread to the other Caribbean countries. Over the last three decades, dengue serotypes 1, 2 and 4 have become endemic in the Caribbean sub-region, producing numerous epidemics at irregular intervals. Co-circulation of these virus types is occurring in many islands such that dengue fever is considered hyperendemic in this region [10].

With the current dengue epidemic in Barbados, where the population's majority are immunologic virgins to this specific serotype, the heightened risk of DHF/DSS acquisition is imminent. Accordingly, over 74.4% of patients were previously infected or had evidence of current exposure in the recent past. Although the actual number of persons with DHF/DSS over the five year period is unknown based on the observation of clinical and laboratory features, at least 4% of patients had suggestive symptoms. This figure gives an average of at least 16 cases of DHF/DSS per year based on the number of patients with IgG antibodies. The ten patients included in the study with known symptoms of DHF/DSS (thrombocytopenia, bleeding gums, haematuria etc), ranged in age from 8 months to 57 years and did not conform to the suggested age range of less than 15 years.

Although the different DENV serotypes can lead to varying clinical and epidemiologic profiles, defining precisely which clinical characteristics are associated with the distinct serotypes has been elusive. Several reports have indicated that DENV-2 and DENV-3 may cause more severe disease than other serotypes and that DENV-4 is responsible for milder illness [11,12]. Certain genotypes within particular serotypes have been associated with epidemics of DHF [13] versus classic dengue, but no correlation with specific clinical features has been reported.

In a Nicaraguan study [14], DENV-2 was associated with greater disease severity, followed by DENV-3, which led to greater hospitalizations in primary illness, while individuals experiencing a primary DENV-2 infection tended to exhibit less notable clinical disease. They also observed that secondary infections were a risk factor for the presence of severe manifestations of dengue in Nicaragua when DENV-2 was the dominant serotype, but not when DENV-1 or DENV-3 predominated. This gives credence to our study, as the number of DHF/DSS occurring yearly was inappreciable, and this can be reflected by the predominant serotype. Also, although, more that 70% of patients were previously exposed due to the detection of IgG antibodies, severe manifestation of the infection were minimal. Previous studies have shown that DENV-3 and DENV-2 were particularly virulent to young children. This observation was corroborated in our study, where numerous infections occurred in those less than 16 years particularly in 2007. These children where immunological virgins and could not mount an appreciable immune response to this infection since most of these children were hospitalized and never have detectable IgG antibodies.

When DENV-1 predominated in the Nicaraguan study, they observed a higher percentage of laboratory confirmations of dengue but the clinical manifestations were milder. Our retrospective analysis has indeed shown that the number of laboratory confirmed cases has decreased particularly in 2005 and this may be attributed to the serotype of dengue present within our population and not reflective of total adherence to preventative methods or a reduction in rainfall. However, there has been a steady increase in infections caused by DENV-3.

## Conclusion

In conclusion, it would therefore be expected that with the predominance of DENV-3 circulating in the population, although this infection may not be severe as previous when DENV-2 predominated, there will be a higher proportion of children being continuously infected with the greater producing severe manifestations. This may overall cause an increase in the apparent number of cases, but with the present system, although more cases are detected with DENV-1 is present, the RT-PCR will enhance the detection particularly where the IgM and IgG antibodies are undetectable by ELISA especially for those samples that under 5 days after symptom onset. This study has essentially characterized the serotypes circulating on the island over the last five years and presents a plausible explanation for the apparent reduction in cases. This study also enhances the surveillance mechanisms for dengue serotype detection and rapid turnaround time particularly for those who may have DHF/DSS. Further characterization of the genotypes is necessary to enhance the epidemiologic profile. Recently, DEN-3/4 composite types have been seen in our population and this could be similar in other Caribbean islands.

## Methods

### Specimen collection

One hundred and seventy-two whole blood specimens collected during the period January 2003 to August 2007 were analyzed for dengue fever infection with onset ranging from 1 to 5 days. These patients were either hospitalized at the Queen Elizabeth Hospital (QEH), Ministry of Health, Barbados, sent through private physicians or through one of the eight (8) outpatient polyclinics on the island. Peripheral blood specimens were separated for serum and stored at -20°C until processing.

### Serology

IgM and IgG antibody-capture ELISAs were performed according to manufacturer's instructions (Focus Diagnostics, Cypress, CA, 90630 USA). Briefly 100 μl of 1:101 diluted serum sample or control sample is added to the washed 96-microwell polystyrene plate previously coated with anti-human antibody specific for IgM or IgG and 100 μl of antigen solution (inactivated lyophilized dengue fever virus antigen with equal amounts of DEN 1–4) was then added. After a 2-hour incubation, followed by washing, 100 μl of affinity-purified and peroxidase mouse anti-flavivirus IgM conjugate was added. Following incubation and washing, 100 μl of substrate (tetramethylbenzidine and hydrogen peroxide) was added. Colour development was stopped with IM sulphuric acid and the OD was read at 450 nm using a microplate autoreader. The levels of specific antibodies were calculated from OD values.

### RT-PCR and amplification

RT-PCR was performed according to manufacturer's instructions. Briefly, RNA was extracted from 280 μl of serum using the QIAamp Viral RNA minikit. Reverse transcription and polymerase chain reaction was performed using the One-Step Superscript III/RT/Platinum Taq Mix (Invitrogen), 0.1 M dithiothreitol (DTT), 5' primer D1 and 3' primer TS1 at a concentration of 0.5 μM each and 3' primers TS2, TS3 and DEN4 at a concentration of 0.25 μM each in a total volume of 50 μl containing 5 μl of RNA. A negative control was included in each run to identify contamination. One cycle of 60°C for 30 min for the reverse transcription was followed by 94°C for 2 minutes, 52°C for 1 min, 60°C for 1 minute and with a final extension of 60°C for 7 minutes [7].

The expected sizes of the amplification products were as follows: 482 bp (DENV-1), 119 bp (DENV-2), 290 bp (DENV-3) and 389 (DENV-4). Ten microlitres of the fifty microlitre mixture was electrophoresed on a 1.5% TAE agarose gel with a 100 bp DNA ladder (Invitrogen).

## Competing interests

The authors declare that they have no competing interests.

## Authors' contributions

MGS conceived the study and participated in its designed and coordination and drafted the manuscript. NCG participated in the design and coordination of the study and performed the molecular studies.

## Authors' information

Marquita Gittens-St. Hilaire is a lecturer in microbiology at the University of the West Indies, Cave Hill Campus adjunct Director of the Leptospira Laboratory (a governmental institution). Her research interests include infectious diseases, with primary focus on dengue, leptospirosis and other zoonotic infections. Nicole Clarke Greenidge is a medical laboratory technology at the Leptospira Laboratory. Her research interests include microbiology, surveillance and epidemiology.
